# Functionally Distinct Shed Subpopulations Detected After Magnetic-Activated Cell Sorting of CD71 and CD146

**DOI:** 10.3390/cells14242010

**Published:** 2025-12-17

**Authors:** Marina Miteva, Emilia Karova, Natalia Grancharova, Mirela Marinova-Takorova, Violeta Dogandzhiyska, Krasimir Hristov, Nikolay Ishkitiev, Vanyo Mitev, Evgeniy Aleksiev, Zornitsa Mihaylova

**Affiliations:** 1 Department of Chemistry and Biochemistry, Medical Faculty, Medical University—Sofia, 1431 Sofia, Bulgaria; 2 Department of Conservative Dentistry, Faculty of Dental Medicine, Medical University—Sofia, 1431 Sofia, Bulgariav.dogandjiska@fdm.mu-sofia.bg (V.D.); 3 Department of Pediatric Dentistry, Faculty of Dental Medicine, Medical University—Sofia, 1431 Sofia, Bulgaria; n.grancharova@fdm.mu-sofia.bg (N.G.);; 4 Department of Dental, Oral and Maxillofacial Surgery, Faculty of Dental Medicine, Medical University—Sofia, 1431 Sofia, Bulgaria; e.petkov@fdm.mu-sofia.bg; 5 Research Institute of Innovative Medical Science, Medical University—Sofia, 1431 Sofia, Bulgaria

**Keywords:** SHED, CD71, CD146, mesenchymal stem cells, multilineage differentiation, regenerative medicine

## Abstract

**Highlights:**

**What are the main findings?**

- SHED subpopulations were isolated based on CD71 and CD146 expression using magnetic-activated cell sorting.
- CD71^+^ and CD146^+^ SHED showed differences in osteogenic and adipogenic potential; the chondrogenic potential seemed unaffected but could not be definitively assessed under the 2D conditions employed.

**What are the implication of the main findings?**

- The marker-based separation provides an initial indication of functional heterogeneity within SHED populations.
- These observations may help guide future work exploring their potential applications in regenerative dentistry and tissue engineering.

**Abstract:**

Mesenchymal stem cells derived from human exfoliated deciduous teeth (SHED) are a promising source for regenerative therapies due to their multipotency, proliferative capacity, and immunomodulatory properties. The present study aimed to isolate and characterize SHED subpopulations based on CD71 and CD146 expression and evaluate their multilineage differentiation potential. SHED were obtained from pediatric donors and separated into CD71^+^, CD71^−^, CD146^+^, and CD146^−^ fractions using magnetic-activated cell sorting (MACS). CD71^+^/CD71^−^ and CD146^+^/CD146^−^ populations were isolated independently; no simultaneous double sorting for both markers was performed. Immunocytochemistry was employed to confirm the expression of surface and intracellular markers, including STRO-1, CD44, nestin, and vimentin. Multilineage differentiation assays toward osteogenic, adipogenic, and chondrogenic lineages revealed that CD71^+^ cells exhibited reduced osteogenic capacity compared to CD71^−^ cells, whereas CD146^+^ cells showed enhanced osteogenic and adipogenic differentiation. Chondrogenic differentiation seemed unaffected by marker expression under the 2D conditions employed. These results highlight functional heterogeneity within SHED populations and indicate that CD71 and CD146 independently influence differentiation outcomes. The selective enrichment of CD146^+^ SHED may enhance osteogenic and adipogenic regenerative applications, while CD71^+^ subsets may serve as a valuable model for studying proliferation and paracrine effects. Limitations include the use of in vitro differentiation assays and the absence of in vivo validation; additionally, combined CD71/CD146 analysis may further clarify the relationship between metabolic activity and stem/progenitor niche characteristics. Overall, marker-based characterization of SHED subpopulations provides insight into their biological properties and potential utility in targeted cell-based therapeutic strategies.

## 1. Introduction

Multipotent stromal cells (MSCs), commonly referred to as mesenchymal stromal or stem cells, have become a central focus in regenerative medicine due to their remarkable biological versatility. Present in a wide range of fetal and adult tissues, MSCs contribute to tissue homeostasis and repair through their dual biological roles: the capacity for multilineage mesenchymal differentiation and the ability to exert potent immunomodulatory and paracrine effects [1,2,3]. These combined properties position MSCs as a promising platform for next-generation therapeutic strategies addressing degenerative, inflammatory, and immune-mediated disorders.

Dental stem cells (DSCs) represent a tissue-specific subset of MSCs obtained from various dental and peridental structures. Several DSC populations have been identified, including dental pulp stem cells (DPSCs), stem cells from human exfoliated deciduous teeth (SHED), stem cells from the apical papilla (SCAP), periodontal ligament stem cells (PDLSCs), dental follicle stem cells (DFSCs), gingival mesenchymal stem cells (GMSCs), and alveolar bone-derived MSCs (ABMSCs) [4]. Among these, SHED are particularly attractive due to their high proliferative capacity, low tumorigenic risk, broad differentiation potential, and pronounced immunosuppressive activity [5]. SHED are considered biologically distinct from DPSCs [6] and express a characteristic panel of MSC-associated markers, including CD44, CD71, CD146, and STRO-1 [7]. Previous studies further show that SHED proliferate more rapidly than DPSCs and bone marrow MSCs and express elevated levels of growth and differentiation-associated factors, such as bFGF and bone morphogenetic proteins [8,9]. Moreover, SHED demonstrate a strong propensity toward osteogenic and adipogenic differentiation, highlighting their enhanced regenerative potential.

Efficient and reproducible isolation of SHED is essential for their accurate biological characterization and for maximizing their applicability in regenerative dentistry and tissue engineering. The heterogeneity of dental tissues requires isolation methods capable of achieving high purity, viability, and yield while preserving functional properties. Although several strategies exist—including density-gradient separation, differential adhesion, fluorescence-activated cell sorting (FACS), and magnetic-activated cell sorting (MACS)—marker-based isolation remains the most precise approach for defining SHED subpopulations [10].

In this context, the present study investigated a MACS-based isolation strategy targeting the markers CD71 and CD146 to refine SHED purification and advance understanding of their regenerative and immunomodulatory characteristics.

## 2. Materials and Methods

### 2.1. Isolation and Culture of SHED

Exfoliated deciduous teeth (*n* = 3) were obtained from pediatric patients treated at the Faculty of Dental Medicine, Medical University of Sofia, Bulgaria. Written informed consent was obtained from all donors prior to sample collection, in accordance with institutional ethical guidelines. The study was conducted in accordance with the Declaration of Helsinki, and approved by the Medical Science Council of the Medical University of Sofia (protocol code 4770, 11 December 2018). The isolation of stem cells from human exfoliated deciduous teeth (SHED) was performed following established protocols [11]. SHED were isolated from three pediatric donors; the study focused on characterizing subpopulation-specific properties, and inter-donor comparisons were not performed. The cells were cultured in Dulbecco’s modified Eagle medium (DMEM) (Invitrogen, Eugene, OR, USA) supplemented with 10% fetal bovine serum (Sigma-Aldrich, St. Louis, MO, USA), 100 U/mL penicillin, 100 µg/mL streptomycin, and 0.25 µg/mL amphotericin B (Invitrogen). Cultures were maintained under standard conditions until reaching approximately 85–90% confluence, after which the cells were subcultured into vessels with a fourfold larger surface area. All experiments were performed on SHED at passages 2–4.

### 2.2. Antibodies

The following antibodies were used in the present study: mouse monoclonal anti-CD71 (Beckman Coulter International SA, Nyon, Switzerland) conjugated with Fluorescein isothiocyanate (FITC); mouse monoclonal anti-STRO-1 antibody (Abcam, Tokyo, Japan); mouse monoclonal anti-CD146 antibody, anti-Vimentin, anti-Nestin, anti-Dentine SyalPhosphoprotein (DSPP), anti-CD44 (Santa Cruz Biotechnology, Dallas, TX, USA); and anti-alkaline phosphatase (ALP) (Sigma-Aldrich) (all primary antibodies are mice IgG) were used at 1:200 for immunocytochemistry or immunohistochemistry staining. Only anti-CD71 and anti-CD-146 were used for magnetic sorting. The rest of the antibodies were used for immunocytochemical validation. As a secondary antibody, Alexa Fluor 568-conjugated donkey anti-mouse IgG (Abcam) was used to detect mouse primary antibodies. DAPI-nucleic acid stain (Invitrogen, Eugene, OR, USA) was used to stain nuclei. MicroBeads conjugated to monoclonal anti-human CD71 antibodies (isotype mouse IgG2a) (Miltenyi Biotec Inc., San Jose, CA, USA) were used at a concentration of 20 μL/10^5^ cells for magnetic separation. Goat Anti-Mouse IgG MicroBeads (Miltenyi) were used according to the manufacturer’s instructions as a secondary antibody for magnetic separation.

### 2.3. Magnetic Separation of CD71 and CD146 Positive Cells

Once the cultured SHED reached a sufficient quantity (>1 × 10^5^ cells) per T25 flask (TPP^®^, Trasadingen, Switzerland), magnetic separation was performed to isolate CD71^+^ and CD146^+^ cell subpopulations following previously described protocol [12]. Cells were detached using trypsin–EDTA (Lonza, Verviers, Belgium) and processed according to the manufacturer’s instructions (Miltenyi Biotec GmbH, Bergisch Gladbach, Germany). Briefly, harvested cells were first incubated with specific primary antibodies, followed by incubation with secondary antibodies conjugated to magnetic microbeads (Miltenyi Biotec GmbH).

The labeled cell suspension was subsequently passed through a magnetic separation column positioned in the magnetic field of a MiniMACS™ Separator (Miltenyi Biotec GmbH). Unlabeled cells passed through the column, whereas magnetically labeled CD71^+^ and CD146^+^ cells were retained. After removal of the column from the magnetic field, the positive fraction was eluted with fresh culture medium. The isolated cells were then maintained in DMEM, until sufficient numbers were obtained for subsequent analyses. SHED were seeded at a density of 5 × 10^2^ cells/cm^2^ in T25 flasks and cultured under standard conditions (37 °C, 5% CO_2_, humidified atmosphere). The purity of the isolated cell populations was verified by flow cytometric analysis.

Magnetic enrichment for CD71^+^ and CD146^+^ SHED subpopulations was performed on separate cell aliquots. Each marker was processed independently, using distinct samples for primary antibody labeling and subsequent MACS separation, with no sequential or combined labeling steps applied.

### 2.4. Cell Counting for Evaluation of CD71 and CD146 Positive Cells

Cell counting for the evaluation of CD71 and CD146 positive subpopulations was performed immediately after magnetic labeling. Cell numbers were determined using a hemocytometer (Neubauer chamber) (Sigma-Aldrich).

Following magnetic enrichment, cell suspensions were washed once with PBS and resuspended in PBS supplemented with 2% FBS. An aliquot was taken for viability and total cell counts. Viability was assessed by trypan blue exclusion: an equal volume of cell suspension and 0.4% trypan blue solution (Sigma-Aldrich) was mixed (1:1), and cells were counted using a hemocytometer. Viable (unstained) and non-viable (blue) cells were enumerated in at least four independent fields (hemocytometer) or by the instrument’s standard protocol.

Cell concentration (cells/mL) was calculated from the counts and used to determine total viable cells recovered per sample. Viability (%) was calculated as:

Viability (%) = Number of viable cells/Total number of cells × 100.

Absolute yield for each magnetically enriched fraction was reported as the total number of viable cells recovered per flask and as a percentage of the initial input cell number. All counts were performed in triplicate for each biological sample and the results are presented as the mean ± standard deviation (SD). When cell aggregates were observed, samples were gently dissociated by repeated pipetting before counting to ensure accurate enumeration.

In addition to cell counting, the separated SHED fractions were further evaluated by immunocytochemistry, as described in the following section.

### 2.5. Immunocytochemistry

Each isolated SHED fraction was seeded into 24-well culture plates at a density of 5 × 10^3^ cells per well (TPP). Following culture under standard conditions, the cells were fixed with 4% paraformaldehyde (Sigma-Aldrich). Furthermore, the cells were rinsed three times with phosphate-buffered saline (PBS) and blocked with 1% bovine serum albumin (BSA) (Sigma-Aldrich) for 30 min at room temperature. For intracellular marker detection, cell membranes were permeabilized immediately after fixation using 0.05% Tween-20 (ICN Biomedicals Inc., Aurora, OH, USA) for 10 min, followed by treatment with 0.05% Triton X-100 (Calbiochem–Merck, Darmstadt, Germany) for an additional 30 min. The cells were incubated with the respective primary antibodies. After washing with phosphate-buffered saline (PBS), the samples were treated with species-appropriate secondary antibodies conjugated to fluorescent dyes. Between each incubation step, excess antibody was removed by PBS washing to minimize background staining. The labeled cells were visualized using an IN Cell Analyzer 6000 imaging system and IN Cell Analyzer Workstation 3.7.3 software (GE Healthcare, Pittsburgh, PA, USA). Using the imaging system, marker expression was detected and quantified across all cultured samples. Across all conditions and markers, more than 2000 cells per well were analyzed, providing sufficient sample size for robust statistical comparison of marker expression and cell counts between compartments (Supplementary File S1).

Image analysis was performed using a standardized, fully automated analysis pipeline in IN Cell Developer, with a single analysis template applied identically to all images and conditions. No manual adjustments of threshold or segmentation parameters were made between groups.

### 2.6. In Vitro Differentiation of SHED

The multilineage differentiation potential of SHED cultures was evaluated toward osteogenic, chondrogenic, and adipogenic lineages. For osteogenic differentiation, cells were cultured in DMEM supplemented with 10% FBS, 50 µg/mL ascorbic acid, 10 mM β-glycerophosphate, and 100 nM dexamethasone (all Sigma-Aldrich). The induction medium was changed every three days, and mineralized nodule formation was assessed after 21 days of culture. Osteogenic differentiation was confirmed by Alizarin Red S (Sigma-Aldrich) staining, which detects calcium deposits within the extracellular matrix. For chondrogenic differentiation, SHED were maintained in a chondrogenic medium containing high-glucose DMEM supplemented with 1% insulin–transferrin–selenium (ITS) (Gibco Life Technologies Inc., Grand Island, NY, USA), 10 ng/mL transforming growth factor-β (TGF-β) (Sigma-Aldrich), 50 µg/mL ascorbic acid (Sigma-Aldrich), and 100 nM dexamethasone (Sigma-Aldrich). After 21–28 days, chondrogenic differentiation was verified by Alcian Blue staining (Sigma-Aldrich) for sulfated glycosaminoglycans in the extracellular matrix. For adipogenic differentiation, cultures were treated with DMEM supplemented with 10% FBS, 0.5 mM isobutylmethylxanthine (IBMX), 1 µM dexamethasone, 10 µg/mL insulin, and 100 µM indomethacin. The differentiation media were replaced every 2–3 days, and lipid droplet formation was observed after approximately 14 days. Adipogenic differentiation was confirmed by Oil Red O (all Sigma-Aldrich) staining, which visualizes intracellular lipid vesicles. All samples were fixed with paraformaldehyde prior staining.

### 2.7. Quantitative Analysis of Alizarin Red S, Oil Red O, and Alcian Blue Staining

Osteogenic, adipogenic, and chondrogenic differentiation of magnetically separated SHED subpopulations (CD71^+^, CD71^−^, CD146^+^, CD146^−^) was quantified using Alizarin Red S, Oil Red O, and Alcian Blue staining, respectively. Bound dyes were extracted from stained samples, and absorbance was measured spectrophotometrically on a microplate reader (Varioskan, Thermo Fisher, Waltham, MA, USA). Values were normalized to total cell number.

#### 2.7.1. Alizarin Red S (Calcium Deposition)

After 21 days of osteogenic differentiation, cells were fixed, stained with Alizarin Red S, and washed thoroughly. The bound dye was extracted with 10% acetic acid for 30 min under gentle agitation, and absorbance was measured at 570 nm. All absorbance values (Alizarin Red S, Oil Red O, and Alcian Blue) were normalized to the total number of cells per well, determined prior to the induction of differentiation.

#### 2.7.2. Oil Red O (Lipid Accumulation)

Following 21 days of adipogenic differentiation, cells were fixed and stained with Oil Red O. The bound dye was eluted with isopropanol and absorbance was read at 510 nm.

#### 2.7.3. Alcian Blue (Glycosaminoglycan Accumulation)

After 21 days of chondrogenic differentiation, cells were stained with Alcian Blue. The dye was extracted with 10% acetic acid or 0.1 N HCl, and absorbance was measured at 620 nm.

### 2.8. Statistical Analysis

All quantitative data are presented as mean ± standard deviation (SD) from three independent biological replicates (*n* = 3). Statistical analyses were performed using one-way ANOVA to evaluate differences among the experimental groups followed by Tukey’s post hoc test when applicable. A *p*-value < 0.05 was considered statistically significant. For analyses involving multiple marker–metric comparisons, Bonferroni adjustment was applied to provide a conservative estimate of significance; however, results are interpreted cautiously given the exploratory nature of the study and the small sample size. All statistical analyses were conducted using IBM SPSS Statistics software (version 31.0.1.0, IBM Corp., Armonk, NY, USA). All analyses were performed on three biological replicates (three donors), with each assay measured in technical triplicates.

## 3. Results

### 3.1. Cell Counting

**Figure 1:**
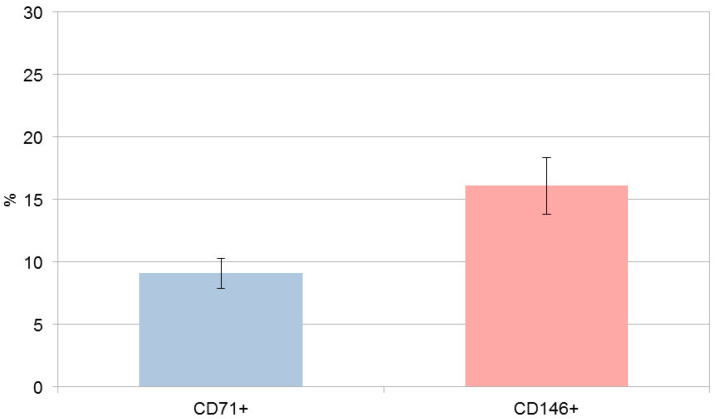
Percentage of CD71^+^ and CD146^+^ SHED after magnetic separation. Data represent mean ± SD of post-MACS separation percentages from three biological replicates, suggesting effective enrichment. Data for other general multipotent markers were not collected in this study.

Cell count evaluation was performed using SHED cultures initially containing approximately 9 × 10^5^ cells per 75 cm^2^ flask prior to marker-based magnetic labeling and separation. Approximately 9.1% (±1.21) of the total SHED population expressed CD71, while 16.1% (±2.29) expressed CD146, indicating the presence of marker-defined subfractions within the SHED culture (Figure 1). CD71^+^/CD71^−^ and CD146^+^/CD146^−^ populations were isolated in separate SHED aliquots; no sequential or simultaneous double sorting was performed. Following magnetic separation, the four subpopulations (CD71^+^, CD71^−^, CD146^+^, and CD146^−^) were expanded to obtain comparable cell numbers for subsequent analyses.

**Figure 2:**
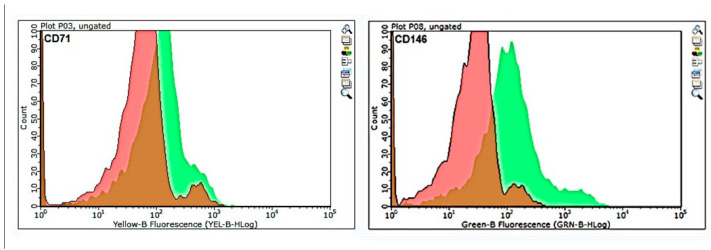
Flow cytometry analysis of CD71 and CD146 expression in SHED prior to magnetic separation (pre-MACS). Histograms show marker expression (green) overlaid with unstained control (red). Approximately 7.8% (±1.7) of the total SHED population was CD71-positive, while 13.6% (±2.4) was CD146-positive. Data represent the pre-MACS population used for subsequent magnetic separation. Experiments were performed in triplicate.

Flow cytometry analysis of the pre-MACS population (Figure 2) indicated that CD146-positive cells were efficiently separated, whereas CD71-positive cells showed partial overlap with the unstained control, suggesting lower enrichment efficiency for CD71-based magnetic separation.

### 3.2. Marker Expression

**Figure 3:**
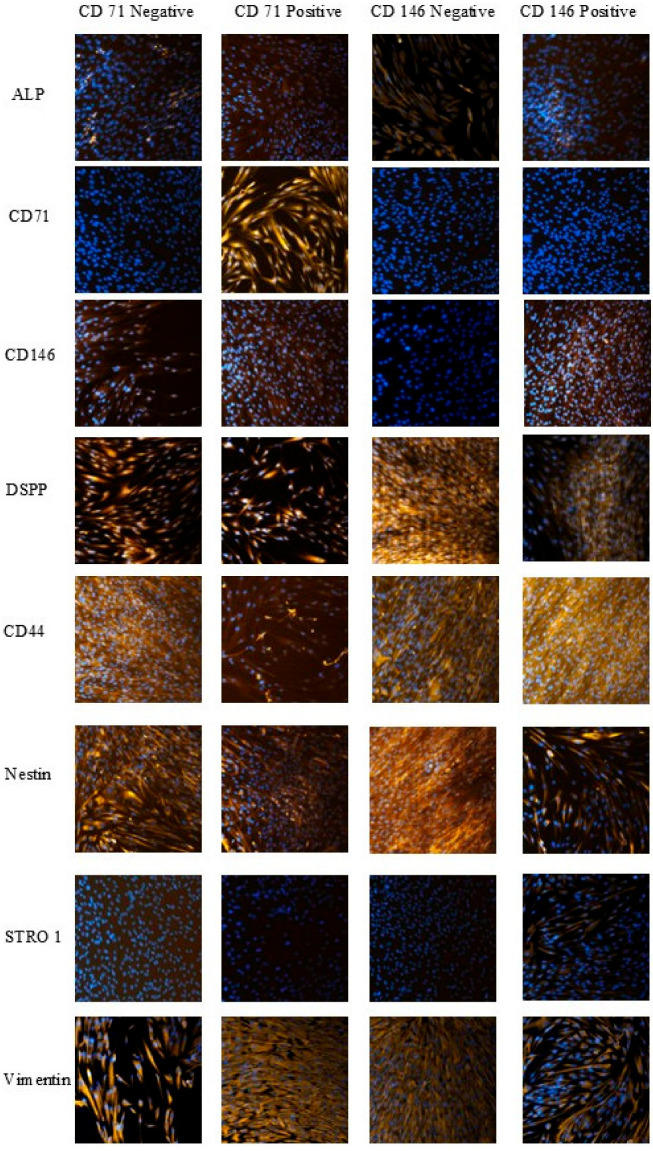
Immunocytochemical analysis of marker expression in isolated SHED subpopulations. Representative images show CD71^+^, CD71^−^, CD146^+^, and CD146^−^ SHED stained for key surface and intracellular markers. Quantitative analysis of fluorescence intensity and cell counts per field confirmed successful enrichment of each marker. Nuclei were counterstained with DAPI (blue). Magnification ×20.

Marker expression in the isolated SHED subpopulations was confirmed by immunocytochemistry, with more than 2000 cells analyzed per condition, enabling robust statistical comparisons of cell abundance and fluorescence intensity. Cells were analyzed for the expression of eight markers (ALP, CD71, CD146, DSPP, CD44, Nestin, STRO-1, Vimentin) across independently sorted CD146-positive/negative and CD71-positive/negative niches (Figure 3). Immunocytochemistry confirmed expected marker enrichment in CD71^+^ and CD146^+^ populations, consistent with successful MACS separation.

CD146-negative cells were more abundant in the ALP-expressing population (486 ± 73 cells/field) than CD146-positive cells (436 ± 98 cells/field; *p* < 0.001). CD71-positive cells exhibited a 73.6% lower proportion of CD44-expressing cells (105 ± 15 vs. 398 ± 37 cells/field; *p* < 0.0001), and DSPP-expressing CD146-positive cells showed reduced abundance relative to CD146-negative cells (168 ± 35 vs. 450 ± 43 cells/field; *p* < 0.0001). These trends indicate that CD71 and CD146 enrich for SHED subsets with distinct marker expression patterns.

Cell-level fluorescence intensity analysis further supported these observations. CD146-negative cells showed the highest ALP and CD44 intensities (1.69-fold and 1.30-fold higher than CD146-positive cells; *p* < 0.0001). CD71-positive cells displayed higher ALP intensity than CD71-negative cells (411.61 ± 34.51 vs. 315.16 ± 20.93; *p* < 0.0001). Nestin was elevated in CD146-positive cells (*p* < 0.01), and Vimentin intensity was lowest in CD71-negative cells.

To verify fluorescence measurements, cell-to-background and nuclei-to-background intensity ratios were calculated for each field by dividing the mean signal within segmented cell or nuclear regions by the mean intensity of adjacent cell-free background. Ratios consistently greater than 1 confirmed that quantified signals exceeded background noise for all markers and conditions.

Taken together, these results suggest the presence of distinct marker-enriched SHED subsets arising from the separate CD71 and CD146 separations. However, as separations were performed independently, any inferred combined CD71/CD146 niche characteristics remain hypothetical.

### 3.3. Cell Morphology and Metabolic State Correlation

Morphometric analysis revealed niche-associated phenotypic differences.

CD71-positive cells were slightly more elongated than CD71-negative cells (*p* < 0.05), whereas CD146-negative cells exhibited compact morphology consistent with higher ALP expression. Cell areas were generally larger in CD71-positive and CD146-negative populations than in CD146-positive cells (*p* < 0.0001). Signal-to-background ratios were highest in CD146-negative and CD71-positive cells, while CD146-positive cells showed lower ratios, possibly influenced by autofluorescence.

These findings indicate associations between marker expression and morphological features but do not establish functional causality.

### 3.4. Statistical Robustness and Effect Size Magnitude

Across 48 comparisons per separation method (8 markers × 6 metrics), both CD146 and CD71 separations showed 31 significant comparisons (64.6%; *p* < 0.05) and 31 large effect sizes (|d| > 0.8). Several differences remained significant after Bonferroni correction (adjusted α = 0.001), indicating consistent trends across markers. The largest effect sizes were observed in Vimentin nuclear area (d = 6.60), DSPP cell area (d = −6.95), and CD146 nuclear area (d = 5.32).

Given the exploratory nature of the study and the limited sample size, these statistical observations should be interpreted cautiously but suggest reproducible patterns between independently sorted SHED subpopulations.

### 3.5. Biological Model Emerging from Integrated Analysis

Integration of abundance, intensity, and morphological parameters revealed parallel trends in the independently sorted CD71 and CD146 fractions. Based on these observations, we propose a preliminary, inferential model of four putative SHED niches. This model is conceptual, as double sorting was not performed:

CD146^−^/CD71^−^: enriched for ALP/CD44 expression and larger, compact cells.

CD146^−^/CD71^+^: showing increased ALP intensity and more elongated morphology.

CD146^+^/CD71^−^: enriched for Nestin and displaying characteristics consistent with perivascular-like phenotypes.

CD146^+^/CD71^+^: a rare group with intermediate marker patterns.

The distributions and marker patterns observed in the independent CD146 and CD71 separations suggest the presence of these four distinct functional cell niches.

### 3.6. Cell Differentiation

**Figure 4:**
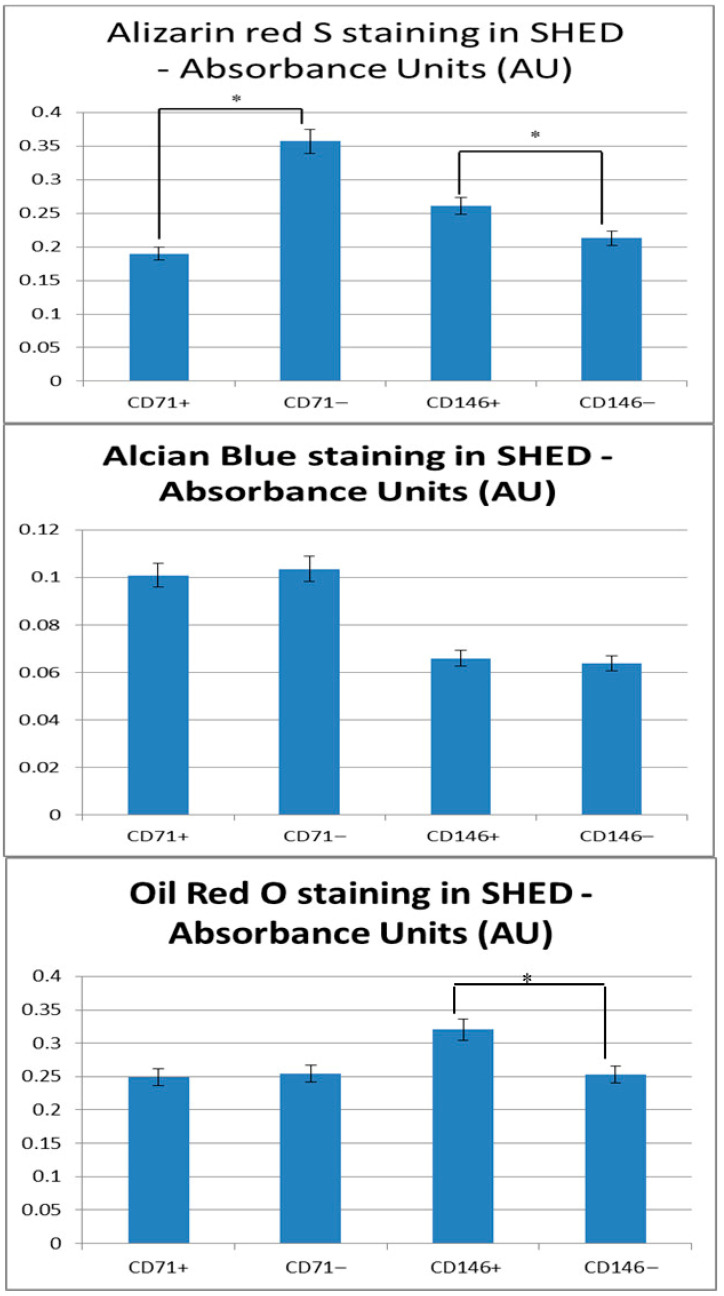
Quantitative analysis of multilineage differentiation in SHED subpopulations. Bar graphs represent osteogenic, chondrogenic, and adipogenic differentiation of CD71^+^, CD71^−^, CD146^+^, and CD146^−^ SHED, assessed by Alizarin Red S, Alcian Blue, and Oil Red O staining, respectively. Data are shown as the mean ± SD of absorbance values. * *p* < 0.01 versus corresponding negative subpopulation. CD71^+^ cells exhibited significantly lower osteogenic differentiation compared to CD71^−^ SHED, whereas CD146^+^ cells demonstrated enhanced osteogenic and adipogenic differentiation relative to CD146^−^ cells. Chondrogenic differentiation showed no significant differences among the subpopulations; note that a 2D monolayer culture was used, which may limit the detection of intrinsic chondrogenic potential. CD71 and CD146 separations were performed independently, ensuring observed effects reflect marker-specific subpopulation properties.

Multilineage differentiation was evaluated across CD71- and CD146-enriched subsets (Figure 4, Supplementary Figures S1 and S2).

Alizarin Red S staining showed reduced mineralization in CD71^+^ compared with CD71^−^ subpopulations (*p* = 0.00299). CD146^+^ SHED demonstrated higher mineral deposition relative to CD146^−^ cells (*p* = 0.000578). Since ALP reflects early differentiation whereas Alizarin Red indicates late mineralization, these differences likely represent stage-dependent patterns rather than contradictory findings. These associations suggest differential osteogenic responses linked to CD71 and CD146 expression.

Chondrogenic differentiation: Alcian Blue staining revealed no significant differences between CD71^+^ and CD71^−^ SHED (0.101 ± 0.001 vs. 0.104 ± 0.002; *p* = 0.057) or between CD146^+^ and CD146^−^ cells (0.066 ± 0.002 vs. 0.064 ± 0.001; *p* = 0.321). It should be noted that this assessment was performed using a 2D monolayer culture, which may not fully support chondrogenesis, and thus these results likely reflect methodological limitations rather than intrinsic differences in SHED subpopulation potential.

Adipogenic differentiation: Oil Red O staining showed comparable lipid accumulation in CD71^+^ and CD71^−^ SHED (0.249 ± 0.012 vs. 0.254 ± 0.004; *p* = 0.618), whereas CD146^+^ SHED exhibited significantly higher adipogenic differentiation than CD146^−^ cells (0.320 ± 0.002 vs. 0.253 ± 0.005; *p* = 0.00266).

Overall, these findings suggest that CD71 and CD146 selectively and independently influence SHED differentiation, with pronounced effects on osteogenic and adipogenic lineages, while chondrogenesis remains unaffected. All separations were performed independently for each marker, and interpretations reflect marker-specific enrichment rather than combined CD71/CD146 phenotypes.

## 4. Discussion

### 4.1. Marker Expression and Population Heterogeneity

The proportions of SHED expressing CD71 and CD146 markers in the present study align with previously reported data, where these antigens are typically detected in 8–15% of the SHED population, with variation dependent on passage number and culture conditions [13,14]. This marker distribution likely reflects the underlying heterogeneity of SHED cultures, which contain functionally distinct subpopulations with differential proliferative and differentiation potentials that may have implications for regenerative applications. To comprehensively characterize this heterogeneity, we analyzed a panel of surface and intracellular markers associated with proliferation, stemness, and odontogenic differentiation, revealing complex patterns of marker co-expression and functional specialization. A limitation of the present study is the absence of an unsorted SHED control. Therefore, while observed differences between CD71^+^/^−^ and CD146^+^/^−^ subpopulations are consistent with marker-specific functional characteristics, we cannot fully exclude potential contributions from the MACS separation procedure itself. Future studies including unsorted SHED controls would be valuable to more definitively rule out such effects.

CD71 (transferrin receptor-1) was detected in a subset of the SHED population, consistent with its established role in iron uptake and active cellular proliferation within mesenchymal stem and progenitor cell populations [15,16]. The identification of CD71^+^ cells suggests the presence of progenitor subsets with elevated metabolic activity and proliferative capacity, yet our immunofluorescence findings paradoxically demonstrate that these metabolically active cells exhibit reduced osteogenic differentiation potential compared to their inactive counterparts. This apparent contradiction underscores the complex nature of functional heterogeneity within SHED populations, where metabolic activity and osteogenic commitment may represent distinct, sometimes opposing, cellular properties.

CD146 (MCAM), a perivascular adhesion molecule characteristically expressed on multipotent MSCs, was present in our SHED cultures and supports previous observations that CD146^+^ cells exhibit enhanced differentiation potential [17]. The association of CD146 with perivascular niches known to maintain stemness suggests that CD146^+^ SHED may constitute a more primitive progenitor niche possessing superior osteogenic and adipogenic capabilities. Similarly, STRO-1, an established marker of early mesenchymal stem cells, was expressed within a discrete subset of SHED, further supporting the presence of primitive progenitor cells throughout the population [18]. The co-expression of CD44, a hyaluronan-binding glycoprotein essential for cell adhesion and migration, confirms the mesenchymal nature of the isolated cells [19]. Additionally, strong expression of the intermediate filament proteins nestin and vimentin reflected the undifferentiated, multipotent phenotype characteristic of mesenchymal stem cells, with Nestin serving as a recognized marker of neural crest-derived progenitors and Vimentin indicating structural integrity and cytoskeletal organization within the heterogeneous mesenchymal culture [20,21].

### 4.2. Stem Cell Function Assessment Following Magnetic Separation

It is important to emphasize that in the present study, CD71^+^/CD71^−^ and CD146^+^/CD146^−^ populations were isolated in separate SHED aliquots using two independent MACS procedures. Sequential double sorting was not performed, therefore, all interpretations regarding combined CD71/CD146 profiles are inferred from parallel analyses rather than directly isolated double-positive or double-negative populations. Analysis of eight markers revealed distinct SHED subsets with divergent phenotypic signatures. Immunocytochemistry confirmed that MACS separation enriched populations consistent with expected antigen profiles. Following separation, substantial differences in cell abundance were observed: CD71^+^ cells exhibited a 73.6% reduction in CD44^+^ stem-like cells, while CD146^+^ cells showed a 62.7% reduction in DSPP-expressing odontogenic cells. These findings indicate that CD71 and CD146 effectively segregate SHED into biologically distinct groups.

Osteogenic patterns differed substantially among subsets. CD146-negative cells displayed the highest ALP and CD44 expression, consistent with reports associating CD146 with endothelial identity and reduced osteogenic commitment [22]. Unexpectedly, CD71-positive cells exhibited higher ALP expression than CD71-negative cells, which may reflect an iron-dependent metabolic osteogenic pathway, suggesting that metabolically active SHED can engage in distinct modes of osteoblast differentiation [23].

CD146-positive cells exhibited significantly elevated Nestin expression and characteristics consistent with a perivascular neural progenitor niche, similar to perivascular structures in dental pulp [24]. Their restricted osteogenic profile supports the existence of a lineage-biased progenitor group influenced by microenvironmental and developmental factors.

### 4.3. Morphological Characteristics and Metabolic Signatures

Cell morphology reflected metabolic and differentiation states. CD71-positive cells, which are metabolically active, showed significantly greater elongation compared to CD71-negative cells, despite similar cell areas, consistent with early differentiation or migratory activity. CD146-negative cells, exhibiting the highest ALP expression, displayed the most compact morphology, indicative of committed osteogenic maturation. Cell areas were largest in CD71-positive and CD146-negative populations, correlating with metabolic activity and differentiation status. Signal-to-background ratios were highest in these same subsets, reflecting robust marker expression, whereas CD146-positive cells showed lower signal, consistent with a quiescent or endothelial-like phenotype.

### 4.4. Integrated Model of SHED Organization

Because CD71 and CD146 separations were performed independently on different SHED aliquots, the proposed CD71/CD146 combinatorial niche should be regarded as an integrative, hypothesis-generating model, rather than as experimentally isolated double-marker populations. This conceptual framework synthesizes parallel trends observed across independently sorted CD71 and CD146 groups and should be interpreted as exploratory.

Within this interpretive model, four functional SHED niches can be conceptualized: CD146^−^/CD71^−^: A committed osteogenic group characterized by maximal ALP and CD44 expression, compact osteoblast-like morphology, and high abundance. CD146^−^/CD71^+^: A metabolically active osteogenic subset with elevated ALP expression, elongated morphology, and iron-dependent metabolic activation. CD146^+^/CD71^−^: A perivascular progenitor-like group with strong Nestin expression, endothelial features, and low metabolic activity. CD146^+^/CD71^+^: A rare cell niche with hybrid endothelial/mesenchymal characteristics and reduced progenitor marker expression.

The convergence of trends across independent CD71 and CD146 enrichments supports this organizational model as a useful exploratory framework for understanding SHED heterogeneity.

### 4.5. Differentiation Potential and Lineage Commitment

To evaluate the lineage-specific differentiation potential of SHED subpopulations, we analyzed markers associated with odontogenic and osteogenic processes. DSPP, a non-collagenous protein secreted by odontoblasts, served as a specific indicator of odontoblastic lineage commitment [25], whereas ALP expression reflected early stage osteogenic activity, and Alizarin Red S staining captured late-stage mineral deposition, characteristic of MSC differentiation [26,27]. These combined markers confirm the mesenchymal and multipotent nature of SHED, highlighting their inherent capacity for both odontogenic and osteogenic differentiation.

Our results show that SHED subpopulations stratified by CD71 or CD146 expression exhibit distinct differentiation tendencies. CD71^+^ cells demonstrated higher ALP expression but reduced Alizarin Red S mineralization, suggesting that they are metabolically active yet may follow a distinct, early osteogenic pathway. In contrast, CD146^+^ SHED exhibited enhanced late-stage mineralization, indicating a more advanced osteogenic commitment. Morphologically, CD71^+^ cells were more elongated, reflecting active differentiation or migration, whereas CD146^−^ cells, with the highest ALP expression, displayed a compact morphology characteristic of committed osteoblasts. These observations indicate that cellular morphology and metabolic state correlate with differentiation stage and marker expression.

Chondrogenic differentiation appeared unaffected by CD71 or CD146 status, consistent with the use of 2D monolayer culture conditions in this study, which may be suboptimal for fully assessing chondrogenesis [28]. Adipogenic differentiation was enhanced in CD146^+^ cells, while CD71 had minimal influence on lipid accumulation, further supporting the idea that CD146 marks highly multipotent SHED capable of robust osteogenic and adipogenic differentiation.

These findings align with previous studies linking CD146 to multipotent mesenchymal progenitors [29] and are corroborated by recent reports identifying CD146^+^ SHED as a highly potent subpopulation with superior differentiation potential [6]. In line with the International Society for Cellular Therapy minimal criteria, SHED cultures were plastic-adherent, demonstrated trilineage differentiation (osteogenic, adipogenic, chondrogenic), and expressed canonical MSC markers (CD73, CD90, CD105 positive; CD34, CD45, HLA-DR negative) [30], confirming their mesenchymal identity. Collectively, our data indicate that CD71 and CD146 can serve as reliable markers for functionally distinct SHED subpopulations, with differentiation patterns that correspond to both metabolic state and cell morphology.

### 4.6. Limitations of the Study

Several limitations should be considered. First, CD71 and CD146 magnetic separations were performed independently in separate SHED aliquots, and thus double-positive or double-negative populations were not directly isolated. As a result, the four-cell-niche model is hypothesis-generating and based on inferred relationships between separately enriched populations. Second, the study did not include an unsorted SHED control group for differentiation assays, limiting the ability to distinguish marker-associated effects from potential influences of the MACS procedure itself. Third, magnetic separation may achieve lower purity resolution compared to flow cytometry. In particular, CD71-positive cells showed partial overlap with the unstained control in pre-MACS flow cytometry histograms, indicating suboptimal enrichment, whereas CD146-positive cells were efficiently enriched. Although enrichment was confirmed by immunocytochemistry, precise post-sorting purity could not be quantified. Fourth, the study was performed with a limited number of pediatric donors, and donor-to-donor variability may influence marker expression and differentiation outcomes. Fifth, the in vitro differentiation conditions do not fully recapitulate the native SHED microenvironment, particularly for chondrogenic assays, which were performed in monolayer rather than pellet culture. Finally, mechanistic pathways underlying the observed phenotypes were not investigated, and future transcriptomic or proteomic studies are needed to define regulatory networks governing SHED subpopulation behavior.

## 5. Conclusions

In conclusion, understanding how SHED subpopulations differ in their differentiation potential and metabolic state is important for informing future cell-based regenerative strategies. Our findings indicate that marker-based separation using CD71 and CD146 can identify SHED subsets with distinct functional properties, although all separations were performed independently for each marker, and unsorted SHED controls were not included. These results provide a foundation for more targeted studies of SHED heterogeneity and highlight the potential for refining regenerative approaches by selecting functionally specialized subpopulations.

## Data Availability

The original contributions presented in this study are included in the article/Supplementary Materials. Further inquiries can be directed to the corresponding author.

## Supplementary Materials

The following supporting information can be downloaded at: https://www.mdpi.com/article/10.3390/cells14242010/s1, File S1: This Excel file contains the raw quantitative data obtained from the automated immunofluorescence image analysis of SHED fractions. The dataset includes cell counts and marker expression values derived from images acquired using the IN Cell Analyzer 6000 system. Quantification was performed using a fully automated analysis pipeline in IN Cell Developer software, applying identical segmentation and thresholding parameters across all samples and experimental conditions. For each condition and marker, data were collected from more than 2000 cells per well and used for subsequent statistical analyses presented in the main manuscript; Figures S1 and S2: Macroscopic images of multilineage differentiation of SHED. Representative macroscopic images of SHED cultured under osteogenic, adipogenic, and chondrogenic differentiation conditions. Osteogenic differentiation was assessed by Alizarin Red S staining, adipogenic differentiation by Oil Red O staining, and chondrogenic differentiation by Alcian Blue staining.

## Abbreviations

The following abbreviations are used in this manuscript:

ABMSCs	Alveolar Bone-Derived Mesenchymal Stem Cells
ALP	Alkaline Phosphatase
ANOVA	Analysis of Variance
BSA	Bovine Serum Albumin
bFGF	Basic Fibroblast Growth Factor
BMP	Bone Morphogenetic Proteins
CD	Cluster of Differentiation
CD44	Cluster of Differentiation 44
CD71	Transferrin Receptor 1
CD146	Melanoma Cell Adhesion Molecule (MCAM)
CO_2_	Carbon Dioxide
DAPI	4′,6-Diamidino-2-phenylindole
DFSCs	Dental Follicle Stem Cells
DMEM	Dulbecco’s Modified Eagle Medium
DPSCs	Dental Pulp Stem Cells
DSPP	Dentin Sialophosphoprotein
EDTA	Ethylenediaminetetraacetic Acid
FACS	Fluorescence-Activated Cell Sorting
FBS	Fetal Bovine Serum
FITC	Fluorescein Isothiocyanate
GMSCs	Gingival Mesenchymal Stem Cells
HCl	Hydrochloric Acid
IgG	Immunoglobulin G
IBMX	3-Isobutyl-1-methylxanthine
ITS	Insulin–Transferrin–Selenium
MACS	Magnetic-Activated Cell Sorting
MCAM	Melanoma Cell Adhesion Molecule (CD146)
MSCs	Mesenchymal Stem/Stromal Cells
PBS	Phosphate-Buffered Saline
PDLSCs	Periodontal Ligament Stem Cells
SCAP	Stem Cells from the Apical Papilla
SD	Standard Deviation
SHED	Stem Cells from Human Exfoliated Deciduous Teeth
SPSS	Statistical Package for the Social Sciences
STRO-1	Stromal Precursor Antigen-1
TGF-β	Transforming Growth Factor Beta
