# Supplementary figures and images for: Functionally Distinct Shed Subpopulations Detected After Magnetic-Activated Cell Sorting of CD71 and CD146
Source: Cells. 2025 Dec 17;14(24):2010. doi: 10.3390/cells14242010 (PMC12731958; doi:10.3390/cells14242010)

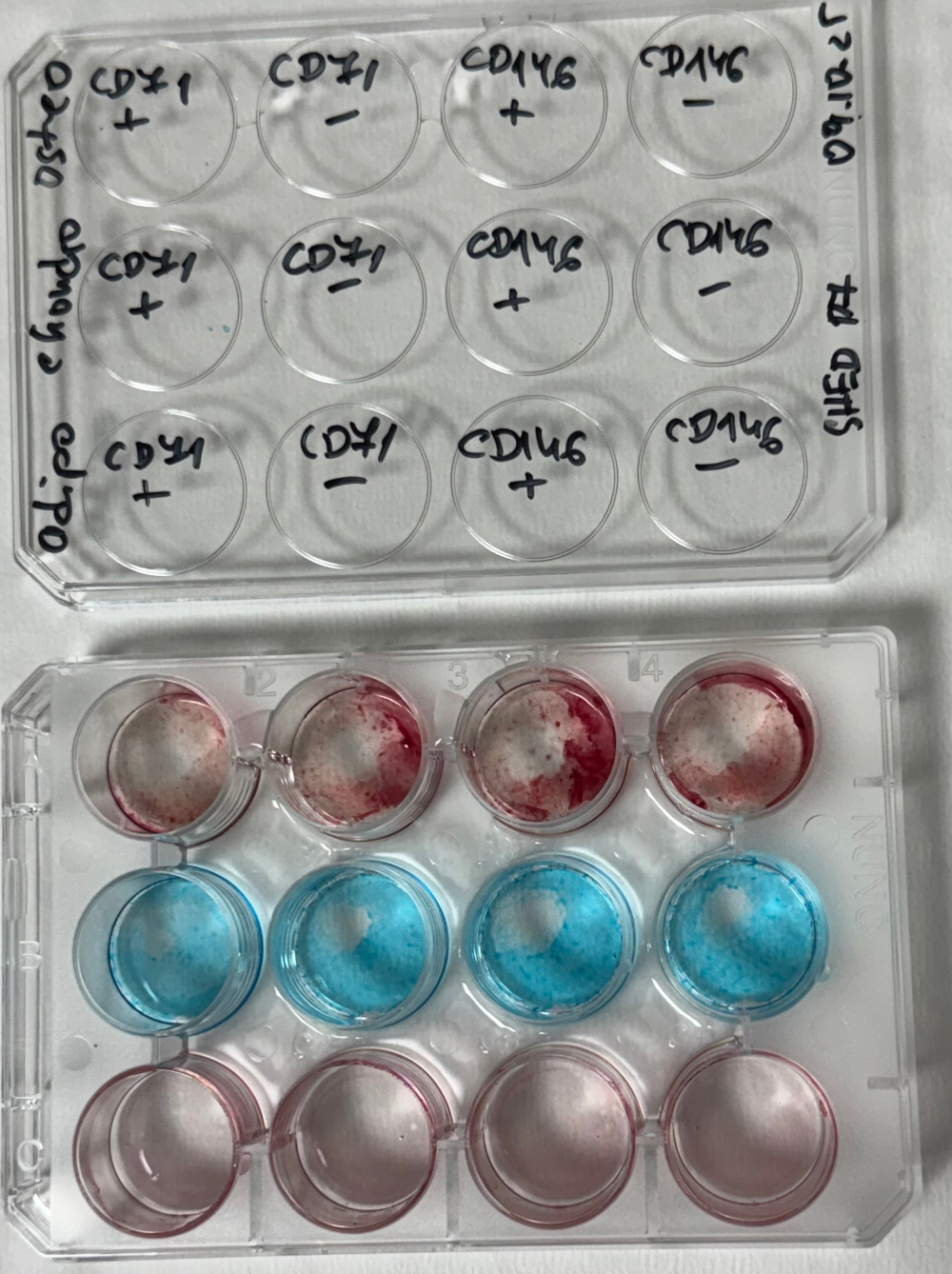

Supplement: Supplementary file 1 [file cells-14-02010-s001.zip › suppl. image1.tif]

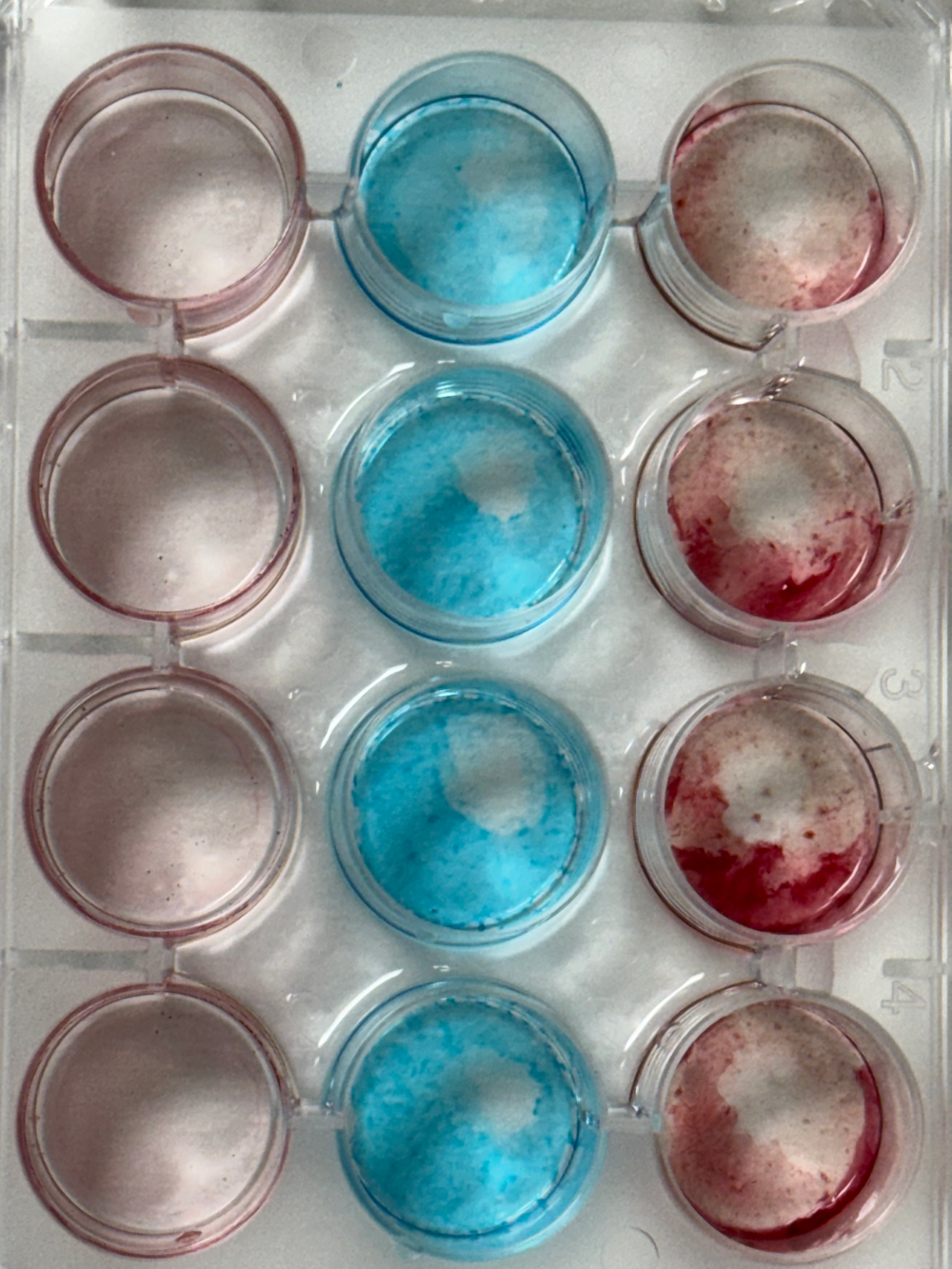

Supplement: Supplementary file 1 [file cells-14-02010-s001.zip › suppl.image2.tif]
